# Multiple Genome Sequences of Heteropolysaccharide-Forming Acetic Acid Bacteria

**DOI:** 10.1128/genomeA.00185-17

**Published:** 2017-04-20

**Authors:** Julia U. Brandt, Frank Jakob, Andreas J. Geissler, Jürgen Behr, Rudi F. Vogel

**Affiliations:** a Technische Universität München, Lehrstuhl für Technische Mikrobiologie, Freising, Germany; b Technische Universität München, Bavarian Biomolecular Mass Spectrometry Center, Freising, Germany

## Abstract

We report here the complete genome sequences of the acetic acid bacteria (AAB) *Acetobacter aceti* TMW 2.1153, *A. persici* TMW 2.1084, and *Neoasaia chiangmaiensis* NBRC 101099, which secrete biotechnologically relevant heteropolysaccharides (HePSs) into their environments. Upon genome sequencing of these AAB strains, the corresponding HePS biosynthesis pathways were identified.

## GENOME ANNOUNCEMENT

Acetic acid bacteria (AAB) are Gram-negative obligate aerobes belonging to the *Alphaproteobacteria* subdivision. They are well known to produce large amounts of exopolysaccharides (EPS)—either homopolysaccharides (HoPSs) such as levans (1–3) and cellulose (4, 5), or heteropolysaccharides (HePSs) such as acetan (6) and gluconacetan (7). HePSs, in particular, have unique properties, since their complex, mostly branched structures are responsible for drastic viscosity increases of aqueous solutions. The food industry is taking increasing advantage of the unique rheological properties of bacterial HePSs. We identified three potential HePS-forming AAB, *Acetobacter aceti* TMW 2.1153, *A. persici* TMW 2.1084, and *Neoasaia chiangmaiensis* NBRC 101099, on sucrose-deficient media. To gain insights into the HePS biosynthesis we sequenced the complete genomes of the identified HePS-producing AAB for further identification of specific HePS biosynthesis clusters. 

*A. aceti* TMW 2.1153 and *A. persici* TMW 2.1084 were isolated from water kefir in our laboratory in Freising, Germany, and *N. chiangmaiensis* NBRC 101099 was isolated from a Thai red ginger flower at the National Institute of Technology and Evaluation Biological Resource Center, Japan (8). High-molecular-weight DNA was purified from modified sodium-gluconate (NaG) medium liquid cultures using the Genomic-tip 100/G kit (Qiagen, Hilden, Germany), as described previously (9). Single-molecule real-time (SMRT) sequencing (PacBio RS II) was carried out at GATC (Konstanz, Germany) (10). A single library was prepared for each of the three strains, and an insert size of 8 to 12 kb was selected for library creation, resulting in at least 200 Mb of raw data from 1 SMRT cell (1 × 120-min movies), applying P4-C2 chemistry. The generated sequences were assembled with SMRT Analysis version 2.2.0.p2 using the Hierarchical Genome Assembly Process version 3 (HGAP3) (11). Initial open reading frame predictions and annotations were accomplished automatically using the NCBI Prokaryotic Genome Annotation Pipeline and Rapid Annotations using Subsystems Technology (RAST), a SEED-based, prokaryotic genome annotation service (12–14).

The genomes were assembled to one circularized chromosome with overall chromosome sizes ranging from 3.23 Mb for *A. persici* TMW 2.1084 to 3.72 Mb for *A. aceti* TMW 2.1153 and G+C contents of 56.83 to 61.50%. *A. persici* TMW 2.1084 harbors an additional plasmid comprising 526,169 bp. The detailed characteristic data, sequencing statistics, genome information, and accession numbers are given in Table 1.

**TABLE 1:** Strain characteristics, sequencing statistics, genome information, and accession numbers

Strain	Source	BioSample no.^a^	Accession no.	Size (Mb)	Coverage (×)^b^	No. of contigs^c^	G+C content (%)	No. of PEGs^d^	No. of CDSs^e^
*A. persici* TMW 2.1084	Water kefir (Freising, Germany)	SAMN 04396916	CP014687 to CP014688	3.23	172	2	57.44	3,387	3,044
*A. aceti* TMW 2.1153	Water kefir (Freising, Germany)	SAMN 04396917	CP014692	3.72	96	1	56.83	3,664	3,200
*N. chiangmaiensis* NBRC 101099	*Alpinia purpurata* (Chiang-Mai, Thailand)	SAMN 04396918	CP014691	3.40	59	1	61.50	3,296	3,002

a All biosamples are part of the BioProject PRJNA311264. Accession numbers are listed for all contigs of each whole genome (as a range).

b Average coverage of HGAP assembly.

c Chromosome plus plasmids and partial plasmids (only the case for TMW 2.1084).

d PEGs, protein-encoding genes (based on RAST annotation).

e CDSs, coding sequences (coding based on NCBI Prokaryotic Genome Annotation Pipeline).

The genome of *A. aceti* TMW 2.1153 harbors three complete rRNA operons (5S, 16S, and 23S) and 52 tRNA genes. *A. persici* TMW 2.1084 encodes 62 tRNAs, and *N. chiangmaiensis* NBRC 101099 encodes 54 tRNAs; both harbor four complete rRNA operons. Among the identified genes, HePS-forming gene clusters could be detected, including *pol* genes of the *polABCDE* cluster associated with pellicle formation (15). Furthermore, in *A. aceti* TMW 2.1153, *gum*-like genes, similar to genes in the *gum* cluster of *Xanthomonas campestris* (16, 17), could be identified. Their accessibility will allow a better investigation of AAB-derived HePSs and the connected biosynthesis, based on specific HePS clusters.

### Accession number(s).

The three complete genomes have been deposited in DDBJ/EMBL/GenBank under the accession numbers given in Table 1.
